# pH-responsive bond as a linker for the release of chemical drugs from RNA–drug complexes in endosome or lysosome

**DOI:** 10.59566/isrnn.2024.0101091

**Published:** 2024

**Authors:** Piotr Rychahou, Sijin Guo, Yinan Zhang, Nicole Rychagov, Yekaterina Y. Zaytseva, Heidi L. Weiss, B. Mark Evers, Peixuan Guo

**Affiliations:** 1 Markey Cancer Center and Department of Surgery, University of Kentucky, Lexington, KY 40536, USA;; 2 Center for RNA Nanobiotechnology and Nanomedicine, The Ohio State University, Columbus, OH, USA;; 3 Comprehensive Cancer Center, The Ohio State University, Columbus, OH, USA;; 4 Nanjing University of Chinese Medicine, Nanjing, Jiangsu, China;; 5 Department of Pharmaceutical Sciences; Center for Pharmaceutical Research and Innovation, University of Kentucky, Lexington, KY 40536, USA;; 6 College of Pharmacy; Center for RNA Nanobiotechnology and Nanomedicine; Comprehensive Cancer Center; Dorothy M. Davis Heart and Lung Research, The Ohio State University, Columbus, OH, USA

**Keywords:** colorectal cancer, RNA nanoparticles, colorectal cancer metastasis, PI3K inhibitors, nanoparticle therapeutics

## Abstract

Emerging phenomena have signaled that RNA therapeutics will be the third milestone in pharmaceutical drug development. RNA nanoparticles display motile and deformable properties that lead to (1) fast and efficient tumor accumulation via both spontaneous and active targeting, and (2) fast renal excretion of non-tumor-accumulated nanoparticles due to RNA’s negative charge and dynamic property; thus, undetectable toxicity. Here, we report the use of RNA nanoparticles to harbor the cancer-targeting ligand and chemical drugs and the design of the pH-responsive bond as a linker for the release of chemical drugs from RNA–drug complexes in endosomes or lysosomes. RNA nanoparticles constructed from a three-way junction (3WJ) core of bacteriophage phi29 packaging RNA (pRNA) offer an advanced strategy for receptor-selective drug delivery into cancer cells and has the potential to enhance the efficacy of anti-cancer therapies while mitigating dose-limiting toxicity in patients with colorectal cancer (CRC). We investigated conjugation of small-molecule drugs targeting the PI3K/mTOR pathway to 3WJ RNA nanoparticles and confirmed intracellular drug delivery by 3WJ RNA nanoparticles into CRC cells. 3WJ a, a single strand of 3WJ nanoparticle, was constructed with a pH-sensitive linkage conjugated to a dual PI3K/mTOR inhibitor, PI-103-azide. We demonstrated PI-103 conjugation to RNA under normal physiologic pH conditions and rapid pH-dependent drug release in an acidic environment. Next, we assembled FA-3WJ-PI103 nanoparticles from three single-stranded RNA, 3WJ a-PI-103, 3WJ b and 3WJ c-folate, to specifically target folate receptor alpha (FRα). Our tests demonstrated receptor-mediated uptake of FA-pRNA-PI-103 nanoparticles, pH-responsive PI-103 drug release from lysosomes and inhibition of the PI3K/mTOR pathway in CRC cells and tumor spheroids. These results confirm receptor-mediated cytosolic drug delivery by pH-responsive RNA nanoparticles and demonstrate potential of the 3WJ–drug complex as a novel strategy for receptor-selective drug delivery to cancer cells with high FRα expression.

## INTRODUCTION

Colorectal cancer (CRC) is the third most common cancer in men and women and the second leading cause of cancer deaths in the United States^1^. Patients with distant metastatic disease have a 5-year relapse-free survival rate ranging from 15% to 35%^2, 3^. Phosphoinositide 3-kinase (PI3K) aberrant signaling from activation or mutations is a frequent event in CRC^4, 5^. The PIK3CA gene encoding the p110α catalytic subunit of PI3K or PTEN, a negative regulator of PI3K, is the most commonly mutated kinase in CRC^5, 6^. PI3K/mTOR is an essential intracellular signaling pathway for cancer cell survival, regulation of cell proliferation and tumor growth^7^. Dual PI3K/mTOR inhibitors combine therapeutic effects by targeting all catalytic subunits of PI3K and both mTORC1 and mTORC2 of the mTOR pathway^8, 9^. However, PI3K/mTOR inhibition comes at a cost of adverse effects, including hyperglycemia, noninfectious pneumonitis and immunosuppression, which significantly limits the use of PI3K/mTOR inhibitors in cancer therapy^7, 10, 11^. Development of carriers for intracellular delivery of small molecule drugs, such as PI3K/mTOR inhibitors, is urgently needed to reduce the frequency of cancer treatment failure due to adverse effects.

The thermodynamically stable 3WJ scaffold provides the necessary modular plasticity to design a carrier for receptor-targeted drug delivery. Each helical branch of the RNA motif can harbor an aptamer or folic acid (FA), which is essential for nanocarrier delivery to cancer cells and minimization of adverse effects associated with small-molecule inhibitors^12, 13^. In this study, we incorporated FA as a moiety against cancer cells with folate receptor alpha (FRα), a membrane-anchored receptor encoded by the FOLR1 gene that is essential for folate transport into cancer cells^14^. Targeting FRα has the following advantages for cancer cell drug delivery: 1) FRα is highly overexpressed in epithelial tumors^15–22^; 2) FRα expression is restricted in normal tissues as most healthy cells use reduced folate carrier (RFC) for folate uptake^23^; 3) when present in normal tissue, FRα is expressed at the apical surface, which is not easily available for exposure to FA-conjugated RNA nanoparticles^23, 24^; 4) cancer cells overexpress FRα on the entire cell surface of ovarian, breast, head and neck, bladder, pancreas, kidney, lung and colon cancers^15–22^, increasing FRα’s access directly from blood circulation; and 5) FRα internalization after nanocarrier binding results in drug delivery into the intracellular space^25^. Programmable properties of RNA, such as defined shape, size, stoichiometry and branch design plasticity, in combination with FRα targeting, provide an attractive tool for cancer cell therapy^26^.

Here, we modified and conjugated PI-103^27–33^, a dual PI3K/mTOR inhibitor, to 3WJ RNA nanoparticles. PI3K/mTOR pathway inhibition resulted in suppression of AKT protein kinase phosphorylation at Th308 and Ser473 residues^34, 35^. Monitoring AKT phosphorylation is a reliable readout of PI3K activity in cancer cells and serves as a confirmation of FRα-mediated drug delivery by 3WJ nanoparticles. We demonstrate that PI-103 remained fully functional and stable over the course of intracellular receptor-targeted small molecule drug delivery into CRC cells. Furthermore, our study confirmed pH-responsive RNA nanoparticles as a viable strategy for receptor-selective drug delivery to cancer cells with high FRα expression, which may offer an effective approach for the treatment of cancers driven by aberrant signaling through PI3K.

## EXPERIMENTAL METHODS

### Cell lines, transfections

HT29 (human colon cancer), SK-OV-3 (human ovarian cancer), JAR (human choriocarcinoma), HCT116 (human colon cancer), LS 174T (human colon cancer) and Caco-2 (human colon cancer) cells were obtained from American Type Culture Collection (Manassas, VA). Cells were previously authenticated by Genetica DNA Laboratories (Cincinnati, OH) and cultured in complete medium purchased from Sigma Aldrich (St. Louis, MO) supplemented with 10% FBS and 1% Gibco antibiotic–antimycotic (Thermo Fisher Scientific, Waltham, MA).

### Western blot

Total protein lysates (20 μg) were resolved on a 4–12% bis-tris gel and transferred to Immobilon PVDF transfer membranes. Membranes were incubated for 40 min at room temperature in blocking solution (TRIS-buffered saline containing 5% nonfat dried milk and 0.1% Tween 20), followed by an overnight incubation in primary antibodies at 4°C. Membranes were then washed 3 times and incubated with horseradish peroxidase-conjugated secondary antibodies for 1 h. After 3 additional washes, the immune complexes on the membranes were visualized by ECL detection. The following antibodies were purchased and utilized in our study: phospho-AKT(Ser473) (Western blot; #4058; 1:1000), Na,K-ATPase (Western blot; #3010; 1:1000), GAPDH (Western blot; #4058; 1:1000), HSP90 (#4877) from Cell Signaling (Danvers, MA). Mouse monoclonal anti-β-actin antibody was obtained from Sigma-Aldrich (St. Louis, MO).

### PI-103-azide preparation for 3WJ conjugation

NaN_3_ (1.43 g, 22 mmol) was added to a solution of (2-chloroethoxy)ethene (2.0 mL, 20 mmol) in DMF (15 mL). The reaction mixture was heated with stirring at 80°C for 4 h. After cooling to room temperature, water (100 mL) and diethyl ether (100 mL) were added to the resulting mixture. The collected organic phase was washed with water (100 mL), brine and dried over Na_2_SO_4_. After removing the volatiles, the residue was purified on silica gel (hexane/EtOAc = 10/1) to provide the product as a volatile colorless oil (600 mg, 27% yield). ^1^H-NMR (CDCl_3_, 400 MHz) δ 6.49 (dd, *J* = 6.8, 14.4 Hz, 1 H), 4.25–4.21 (m, 1H), 4.09–4.08 (m, 1H), 3.88–3.85 (m, 2H), 3.51–3.48 (m, 1H) ppm.

TFA (12 μL, 0.16 mmol) was added to a solution of PI-103 (40 mg, 0.11 mmol) and (2-azidoethoxy)ethane (40 μL, 0.33 mmol) in DCM (5 mL). The reaction mixture was stirred at room temp for 2 days. After removing the volatiles, the residue was purified on silica gel (DCM/MeOH = 70/1–50/1) to provide the product as a colorless oil (20 mg, 40% yield). ^1^H-NMR (CDCl_3_, 400 MHz) δ 8.61–8.57 (m, 2 H), 8.03–8.00 (m, 1H), 7.58–7.54 (m, 1H), 7.39 (t, *J* = 8.0 Hz, 1H), 7.14 (d, *J* = 8.4 Hz, 1H), 5.62 (q, *J* = 5.2 Hz, 1H), 4.04 (s, 4H), 3.79–3.78 (m, 5H), 3.68–3.55 (m, 4H), 3.40–3.39 (m, 3H), 1.45 (d, *J* = 5.2 Hz, 3H). ^13^C NMR (CD_3_OD, 100 MHz) δ 171.6, 167.4, 166.1, 159.3, 157.8, 155.9, 148.7, 142.3, 141.1, 138.9, 130.9, 130.3, 127.9, 126.0, 123.9, 108.4, 75.5 × 2, 73.9, 59.6 × 2, 54.8, 29.5 ppm. HRMS-ESI (m/z): [M+H]^+^ calcd for C_23_H_24_N_7_O_4_, 462.1890; found 462.1885.

### Conjugation of PI-103 to 3WJ nanoparticles

The Folate (FA)-3WJ-PI-103 nanoparticle was assembled from three single-stranded RNA (ssRNA): 3WJ a-PI-103 (5’-PI-103-uuGccAuGuGuAuGuGGG-3’); 3WJ b (5’-cccAcAuAcuuuGuuGAucc-3’); 3WJ c-Folate (5’-Folate-GGAucAAucAuGGcAA-3’) [lower case means 2’ Fluoro (2’-F) modified nucleotide]. 3WJ a-PI-103 was synthesized by conjugating PI-103-azide (PI-103-N_3_) to 5’-alkyne labeled 3WJ a via Copper(I)-catalyzed azide-alkyne cycloaddition (CuAAC). Briefly, 3WJ a-alkyne was synthesized by solid-phase chemical synthesis and using 5’-hexynyl phosphoramidite (Glen Research Corp.; Sterling, VA). 10 μL 1 mM of 3WJ a-alkyne (in diethylpyrocarbonate (DEPC)-treated water) was added to 20 μL 2.5 mM of PI-103-N_3_ (5 eq. in 3:1 (v/v) dimethyl sulfoxide (DMSO) (Acros Organics; Pittsburgh, PA)/tert-Butanol (t-BuOH) (Sigma Aldrich; St. Louis, MO), followed by adding 3 μL 33.3 mM of freshly made “click solution” (a mixture of 0.1 M Copper(I) bromide (CuBr, Sigma Aldrich) with 0.1 M Tris[(1-benzyl-1H-1,2,3-triazol-4-yl)methyl]amine (TBTA, Sigma Aldrich) at a 1:2 molar ratio in 3:1 DMSO/t-BuOH). The reaction was mixed well and run at room temperature for 6 h. The reaction product was evaluated by 20% 8 M urea polyacrylamide gel electrophoresis (PAGE) in TBE (89 mM Tris base-borate, 2 mM EDTA) buffer at 200 V for 1 h. The reaction mixture was subsequently precipitated by adding 1/10 volume of 3 M sodium acetate (NaOAc) and 2.5 volume of ethanol and incubated at −20°C overnight. The precipitates of 3WJ a-PI-103 were resuspended in DEPC-H_2_O and purified through ion-pair reverse phase HPLC on an Agilent PLRP-S 4.6 × 250 mm 300A column. 3WJ b and 3WJ c-Folate strands were purchased from Nanobio Delivery Pharmaceutical Co. (Columbus, OH). Alexa647-labeled 3WJ *b-strand* was purchased from TriLink Bio Technologies (San Diego, CA).

The FA-3WJ-PI-103 nanoparticle was constructed by mixing 3WJ a-PI-103, 3WJ b and 3WJ c-Folate at equal molar concentrations in TMS buffer (50 mM Tris pH 8.0, 100 mM NaCl, 10 mM MgCl_2_), followed by heating to 85°C for 5 min and slowly cooling to 4°C over a 55 min period. The success of assembly was determined by 15% native PAGE in TMS buffer at 120 V for 1 h.

### In vitro acidic pH-triggered PI-103 releasing assay

3WJ a-PI-103 (10 μM) was incubated in a serial saline buffer from pH 8.0 to 3.0 at 37°C for different timepoints. After the entire time course, all samples were evaluated by 20% 8 M urea PAGE in TBE buffer at 200 V for 1 h. The gel bands were quantitatively analyzed by ImageJ and the percentage of PI-103 release was calculated by PI-103 release percent = [(intensity of lower band) / (intensity of upper band + lower band)] × 100%. Upper bands indicate intact 3WJ a-PI-103 while lower bands indicate 3WJ a after PI-103 releasing. The samples with pH = 5.0 and 7.0 buffer treatments were also evaluated by HPLC in an Acetonitrile ramp. The saline buffer pH range from 8.0 to 3.0 was prepared by tittering original saline buffer pH of 8.0 with hydrochloric acid.

### Dynamic light scattering (DLS) assay

FA-3WJ-PI-103 nanoparticles were assembled at 0.2 mM in 50 μL TMS buffer. The average apparent hydrodynamic diameter of FA-3WJ-PI-103 was measured by using Zeta-sizer nano-ZS (Malvern Instruments, Malvern, England) at 25°C with 3 independent measurements. The laser wavelength was 633 nm.

### Temperature gradient gel electrophoresis (TGGE)

FA-3WJ-PI-103 nanoparticles were loaded into a 15% native PAGE and run at 100 V for 10 min at room temperature. TGGE was subsequently performed by applying a gradient temperature (30°C to 80°C) perpendicular to electrical current and run for 60 min at 20 W, as reported previously^36^. T_m_ value was defined as the temperature at which 50% of the nanoparticles dissociated, as quantitatively analyzed by ImageJ.

### Patient-derived xenograft (PDX) engraftment into SCID mice, CRC PDX cell line establishment and CRC experimental metastasis models

NOD-SCID IL2Rgamma^null^ mice were purchased from The Jackson Laboratory (Bar Harbor, ME). Housing for these animals was maintained in a HEPA-filtrated environment within sterilized cages with 12 h light/12 h dark cycles. All animal procedures were conducted with approval of and in compliance with the University of Kentucky Institutional Animal Care and Use Committee. The original patient CRC tumor (F0 generation) was divided and implanted into the flank of a NOD-SCID IL2Rgamma^null^ (The Jackson Laboratory; 005557) mouse. When tumors reached 1 cm^3^ in size, each tumor (F1 generation) was resected, divided into 2 mm^3^ pieces and implanted into 5 mice (F2 generation).

Liberase DH (05401054001; Roche Applied Science, Penzberg, Germany) was resuspended in sterile water to a 2.5 mg/ml concentration and stored in single-use 100 μl aliquots at −80°C. Collagenase/hyaluronidase (07912; StemCell Technologies, Vancouver, BC) was aliquoted into single-use 250 μl aliquots and stored at −80°C. PDX tumors were collected from mice and placed into complete cell culture media supplemented with 1X Gibco^®^ antibiotic–antimycotic (15240–062; Life Technologies, Carlsbad, CA) for transportation. Tumor fragments were minced into 2 mm cubes using scissors and digested in 50 μg/ml Liberase DH (100 μl) and 0.5X collagenase/hyaluronidase (250 μl), diluted in 5 mL of McCoy5A serum free media for 4 h at 37°C with gentle agitation by magnetic stirring bar. No undigested tissue was observed. Digested cells were washed twice with complete cell culture media and transferred into 10% FBS DMEM media supplemented with 1X Gibco^®^ antibiotic–antimycotic and 100 μg/ml primocin (ant-pm-1; InvivoGen, San Diego, CA). Cells harvested from these cultures were injected intravenously into another set of SCID mice.

For intravenous (iv) injection of PDX2387 cells, NOD-SCID IL2Rgamma^null^ mice were anesthetized with isoflurane (induction 4%, maintenance 2%). The viability of cells used for inoculation was greater than 95% as determined by Vi-CELL^™^ XR (Beckman Coulter, Brea, CA); 1 × 10^6^ cells were injected per animal. Gentle pressure was applied to the inoculation site until there was no visible sign of bleeding. Lung tissues were preserved for histological examination by fixation in 10% buffered formalin followed by paraffin embedding.

For intrasplenic (isp) injection of colorectal cancer PDX2377LM cells, NOD-SCID IL2Rgamma^null^ mice were anesthetized with isoflurane (induction 4%, maintenance 2%). The viability of cells used for inoculation was greater than 95% as determined by Vi-CELL^™^ XR (Beckman Coulter); 5 × 10^6^ cells were injected per animal. Liver tissues were preserved for histological examination by fixation in 10% buffered formalin followed by paraffin embedding.

### Spheroid 3D cultures

Tumor spheroids were generated by plating PDX cells in ultra-low attachment 24-well plates (Corning, #CLS3474–24EA); this method stimulated the formation of spheroids within 24 h. RPMI 1640 FA-free medium (#27016021) and dialyzed fetal bovine serum (26400044) were purchased from ThermoFisher Scientific (Waltham, MA). Tumor spheroids were cultured in FA-free complete media (10% dialyzed bovine serum; RPMI 1640 without FA) before treatment with FA-3WJ-PI-103.

### Immunohistochemistry

Immunohistochemistry (IHC) was performed as previously described^34^. FRα IHC Assay Kit was purchased from Biocare Medical (Pacheco, CA). Briefly, slides were deparaffinized in xylene, rehydrated, incubated for 5 min with fresh 0.3% hydrogen peroxide, washed with PBS and heated to 95°C for 1 h in antigen retrieval solution (Diva Decloaker; Biocare Medical, Concord, CA). Slides were cooled to room temperature for 30 min and then washed with PBS. Sections were blocked for 5 min; primary antibody was incubated for 12 h at 4°C, washed with PBS, incubated with secondary antibody (60 min; RT), washed with PBS and incubated with HRP polymer (60 min; RT). All sections were counterstained with hematoxylin and observed by light microscopy. For negative controls, primary antibody was omitted from the above protocol.

### Confocal microscopy

Tissue samples were fixed in 4% paraformaldehyde (Polysciences; #18814; Warrington, PA) with 10% sucrose (Sigma-Aldrich; St. Louis, MO) for 12 h at 4°C and embedded into OCT on dry ice (Tissue-Tek; Andwin Scientific, Schaumburg, IL). Frozen sections of tissues (10 μm thickness) were dried overnight in the dark, washed in RT PBS, stained with Hoechst 33342 (Life Technologies; H21492; 0.5 μg/ml; PBS) nuclear stain and mounted in aqueous ultramount permanent mounting medium overnight (Dako, Glustrop, Denmark; S1964).

### Statistical analysis

All data are presented as the average ± standard error. Significance was determined using one-way analysis of variance (ANOVA) with Holm’s p-value adjustment for multiple comparison correction. *p*<0.05 was considered to indicate a statistically significant difference. Statistical analyses were performed using SAS software ver. 9.4 (SAS Inc., Cary, NC, USA).

## RESULTS

### FA-3WJ binding to cancer cell FRα

**Fig. 1.**
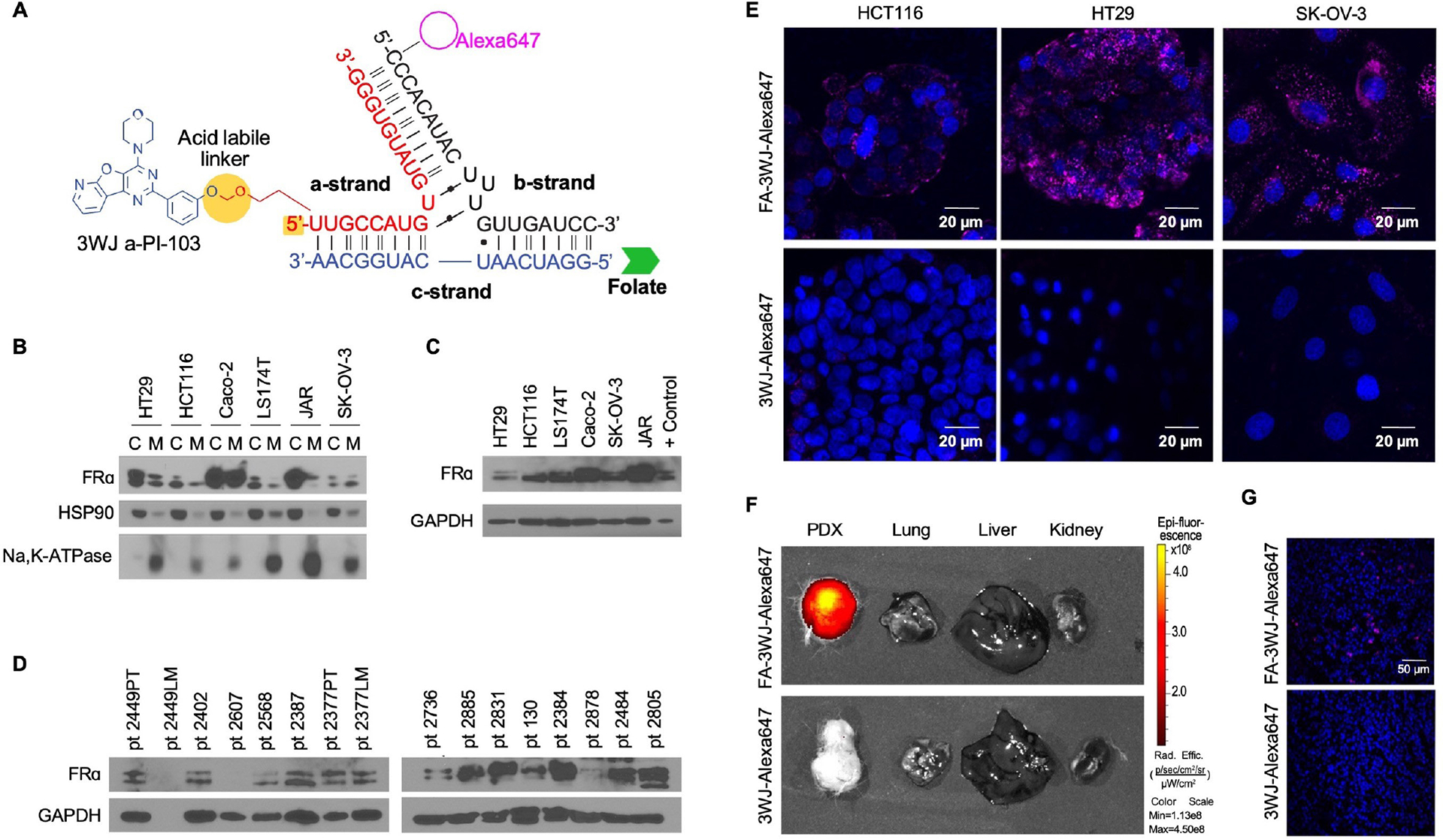
FA-3WJ-Alexa647 binding and internalization in FRα expressing cancer cells lines. **(A)** Illustration of the FA-3WJ-PI-103 assembly for PI3K small molecule inhibitor delivery. **(B)** FRα expression was analyzed in cytoplasmic and membrane fraction of HT29, HCT116, LS174T, Caco2, SK-OV-3, Jar cell lines. **(C)** FRα expression was analyzed in whole-cell protein lysate. **(D)** FRα expression was analyzed by western blot in protein extracts from primary CRC patient tumor samples. **(E)** HCT116, HT29 and SK-OV-3 cells were incubated with FA-3WJ-Alexa647 nanoparticles for 4h in F- 5% FBS cell culture media; binding and internalization of fluorescent nanoparticles was analyzed with confocal microscopy; blue: DAPI stain for nuclear DNA; magenta: Alexa647; magnification 100x. (**F**). NOD SCID mice were transplanted with gen2 PDX 2377LM and placed on a folate free diet. Three weeks after tumor implantation mice were treated with 3WJ-Alexa647 and FA-3WJ-Alexa647 pRNA every two hours (3 treatments; 1.5 μg/g; 500 μl; pRNA diluted in PBS). Two hours after last pRNA injection tumors and organs were collected and imaged on IVIS Spectrum. (**G**). Confocal microscopy of frozen, fixed tissue sections from 2377LM PDX tumors treated with 3WJ-Alexa647 and FA-3WJ-Alexa647 (blue, Dapi; magenta, Alexa647, 20x objective).

Nanoparticle arms of 3WJ are capable of self-assembly out of individual strands that can be modified to carry a specific payload. We have conjugated the *a-strand* to carry the PI3K/mTOR inhibitor, PI-103; the *b-strand* to carry a fluorescent probe for detection of nanoparticle binding to cancer cell surface receptors; and *c-strand* to carry folate for specific binding to cancer cells FRα (Fig. 1A). FRα-mediated RNA nanoparticle binding and entry into cancer cells is an important step in achieving therapeutically effective drug delivery and depends on FRα expression in the target tissue. We analyzed the expression of FRα in CRC cell lines compared to the FRα-overexpressing ovarian cancer cell line, SK-OV-3; a choriocarcinoma cell line, JAR; and in patient-derived xenografts (*n*=16; PDX). High FRα membrane expression was detected in Caco-2 and JAR cell lines. Whole cell extract FRα expression in CRC cell lines was comparable to FRα-overexpressing JAR and SK-OV-3 cell lines (Fig. 1B–D). Next, we tested FA-conjugated fluorescent 3WJ nanoparticle binding in HT29, HCT116 and SK-OV-3 cell lines. Cells were incubated with FA-3WJ-Alexa647 nanoparticles for 4 h in FA-free media and analyzed for binding of fluorescent nanoparticles to FRα by confocal microscopy. FA-3WJ-Alexa647 nanoparticles showed high binding ability to the cell lines with low (HCT116) and high (SK-OV-3 and HT29) FRα expression (Fig. 1E). Finally, we examined FA-3WJ-Alexa647 nanoparticle binding to subcutaneous and metastatic CRC tumors. Two PDX cell lines were developed from CRC liver and lung metastases (i.e., 2377LM and 2387, respectively) (Supplemental Fig. 1A). Mice with subcutaneous 2377LM tumors and 2387 lung metastases were treated with FA-3WJ-Alexa647 and analyzed for fluorescently labeled particle distribution and binding to cancer cells *in vivo*. In both animal models, fluorescent nanoparticles demonstrated no accumulation in normal organs and a strong fluorescent signal either in subcutaneous or metastatic tumors (Fig. 1F, G; Supplemental Fig. 1B). In this section of the study, we have demonstrated *c-strand* conjugation to FA, nanoparticle binding to FRα receptor and high binding specificity to FRα by FA-3WJ nanoparticles.

### PI-103-azide prodrug preparation and conjugation to RNA

**Fig. 2.**
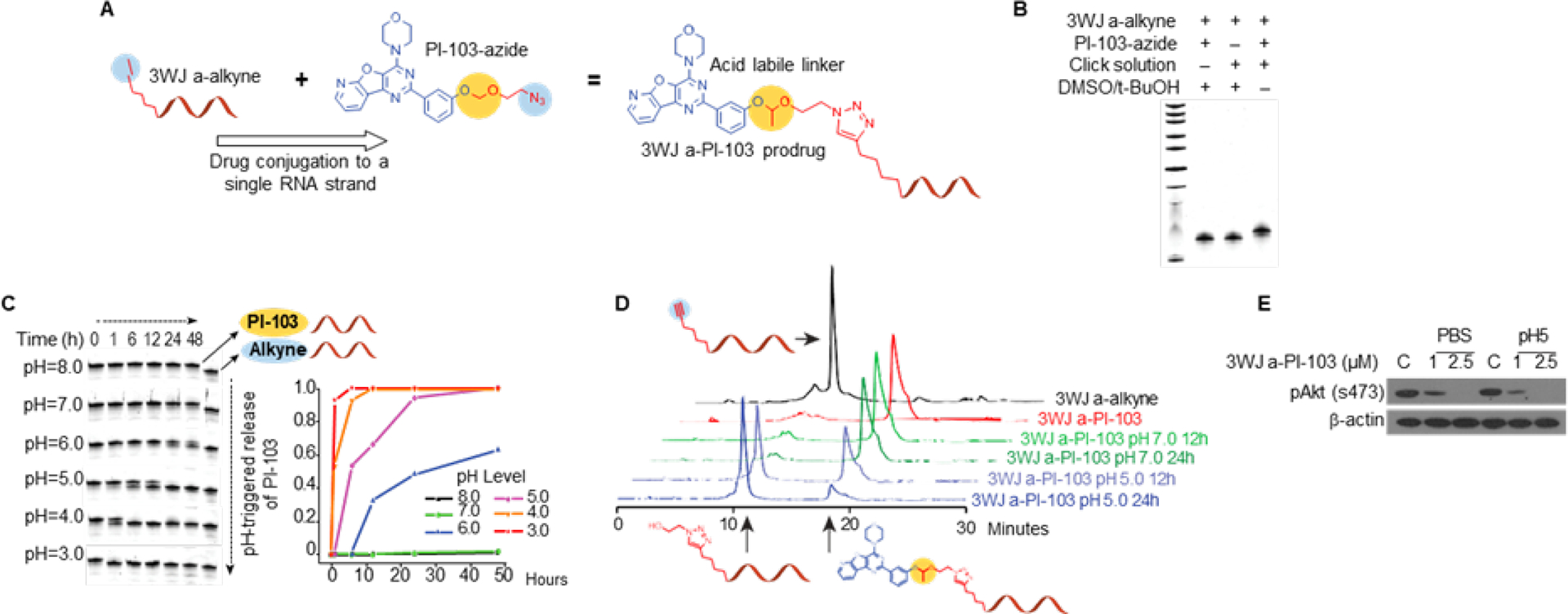
The design of pH-responsive RNA-PI-103 conjugates with acid labile linker. **(A)** PI-103-azide reaction with 5’-end alkyne labeled 3WJ *a-strand* via “click” chemistry to form a 3WJ a-PI-103 prodrug; PI-103 release from RNA strand by hydrolysis at acidic pH. **(B)** Gel assay of 3WJ a-PI-103 conjugation by 20% 8M Urea PAGE. **(C)** In vitro acidic pH-triggered PI-103 releasing profile. **(D)** HPLC spectra of 3WJ a-PI-103 incubated with pH 5.0 and pH 7.0 buffer for 12h and 24h. PI-103 gradually released from 3WJ a-PI103 at pH 5.0 buffer. **(E)** 3WJ a-PI-103 was added to HCT116 cells in complete media (10% FBS) after incubation in 100 μl of pH 5 and pH 7.0 PBS for 4h.

For intracellular drug delivery, we selected the *a-strand* for conjugation to a PI3K/mTOR inhibitor via click chemistry. A PI3K/mTOR inhibitor with available hydroxyl group was used to prepare PI-103-N3 for conjugation to 3WJ a-alkyne, as shown in Supplemental Figs. 2–3. The 3WJ a-alkyne was reacted with PI-103-N_3_ via CuAAC ‘click’ reaction to produce a 3WJ a-PI-103 complex (Fig. 2A). The successful and efficient conjugation was confirmed by the slower migration of 3WJ a-PI-103 in a denaturing PAGE due to the increased molecular size and hydrophobicity from PI-103 compared to 3WJ a-alkyne (Fig. 2B). After conjugation, the poor water-solubility of PI-103 was significantly improved as the 3WJ a-PI-103 was readily dissolved in saline solution. The release of PI-103 from 3WJ a-PI-103-strand can be readily triggered by acidic conditions (Fig. 2C).

When 3WJ a-PI-103 was incubated in acidic pH buffers (≤ 6) over time, the bands of 3WJ a-PI-103 gradually disappeared while the bands of 3WJ a without PI-103 were readily apparent. It can be clearly observed that PI-103 was continuously released from 3WJ *a-strand* when incubated in acidic conditions. The quantitative release curve shows that the PI-103 release rate increased as the pH values decreased, demonstrating an acidic pH-triggered drug release (Fig. 2C, D). Drug release from the *a-strand* or assembled 3WJ nanoparticle and following PI3K inhibition can be used as a sensitive tool to confirm intracellular lysosomal drug release and lysosomal drug escape since PI3K inhibition can be detected within hours after nanoparticle delivery. Therefore, we examined whether drug release from 3WJ *a-PI-103* strand in a low pH environment could result in PI3K/mTOR pathway inhibition. The 3WJ a-PI-103 was incubated in 100 μl of pH 5 and pH 7 PBS for 4 h and added to HCT116 cells in 500 μl of FA-free complete media at 1 and 2.5 μM final concentration for 4 h. Cells were collected and analyzed for pAKT(Ser473) activation, a marker of PI3K inhibition. Our results demonstrate successful drug release from the *a-strand* of 3WJ and PI3K pathway inhibition. In addition, we observed PI3K pathway inhibition in a neutral pH group, which suggests that *a-strand* is degraded after addition to the nuclease-rich environment of complete media (Fig. 2E). This is an important observation of single-strand RNA carrier instability and how the 3WJ motif, when assembled from three pieces of RNA strands, transforms into a structure with high thermodynamic stability, resistance to denaturation even in the presence of 8 M urea and stability at ultra-low concentrations^37^.

### Characterization of fully assembled FA-3WJ-PI-103 nanoparticles *ex vivo*

**Fig. 3.**
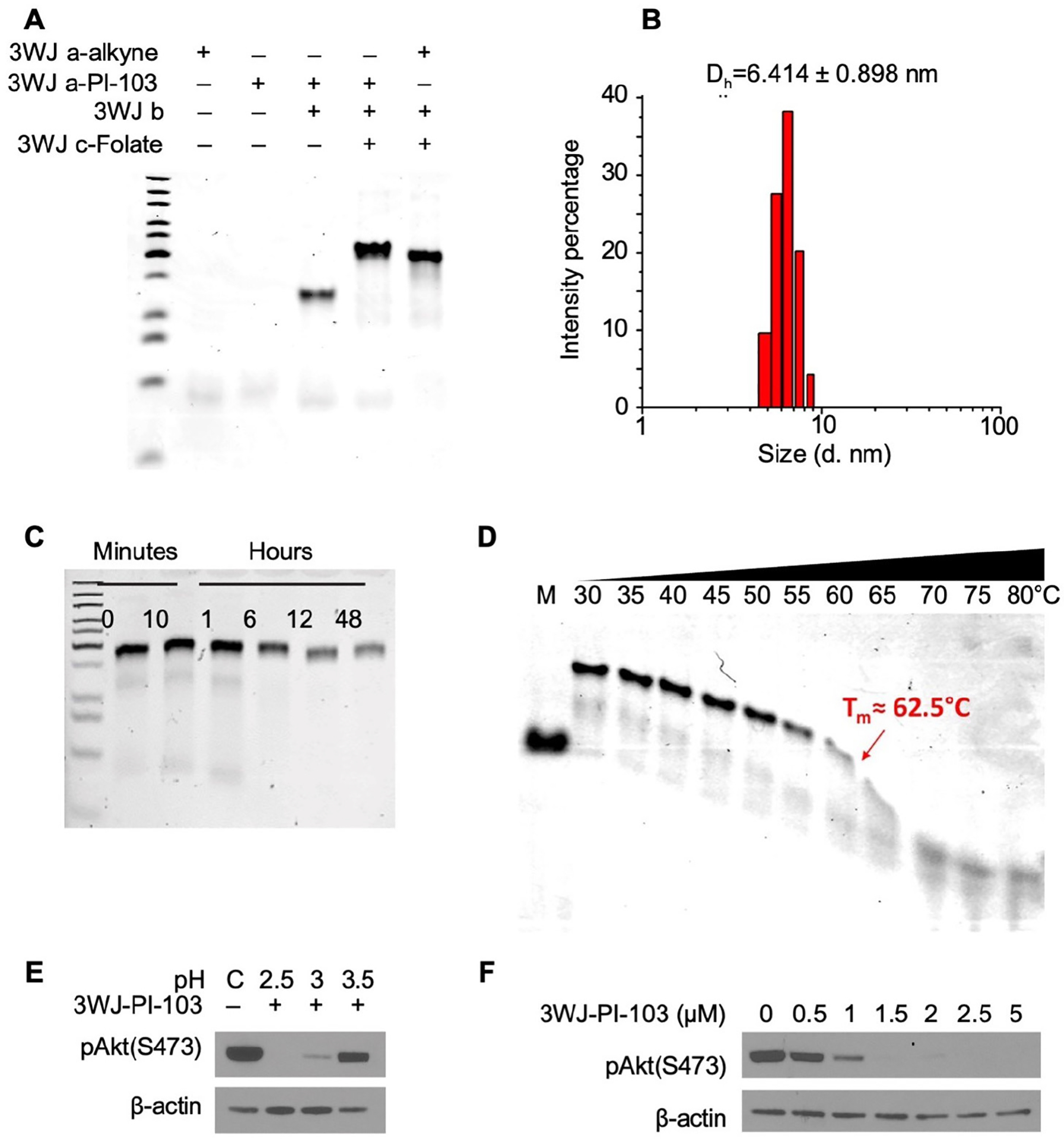
pH-dependent drug release and serum stability from fully assembled 3WJ-PI-103 nanoparticles. **(A)** Stepwise self-assembly of FA-3WJ-PI-103 evaluated by 15% native PAGE. **(B)** The average hydrodynamic diameters of FA-3WJ-PI-103 measured by DLS. **(C)** Serum stability assay of FA-3WJ-PI-103 conjugates in cell culture medium (pH 7.5) containing 10% FBS. (**D**). T_m_ measurement of FA-3WJ-PI-103 by TGGE. **(E)** Western blot analysis of PI3K inhibition after PI-103 drug release at various pH. 3WJ-PI-103 nanoparticle was incubated in 100 μl pH 2.5, 3, 3.5 PBS for 4h and added to HCT116 cells in 400 μl of F- complete media at 5 μM concentration for 4h. **(F)** Western blot analysis of PI3K inhibition after PI-103 drug release at various incubation timepoints. 3WJ-PI-103 nanoparticle was incubated in 100 μl of pH 3 PBS for 4h and added to HCT116 cells in 400 μl of F- complete media at 0.5, 1, 1.5, 2, 2.5 and 5 μM concentration for 4h.

The FA-3WJ-PI-103 nanoparticles were assembled from three RNA fragments (3WJ a-PI-103, 3WJ b and 3WJ c-FA) via a bottom-up self-assembly approach. The assembly was extremely efficient, as few ssRNA fragments were detected in native PAGE, and construction of RNA nanoparticles was not hindered by 5’-end conjugated PI-103 (Fig. 3A). The average hydrodynamic diameter of FA-3WJ-PI-103 was determined to be 6.414 ± 0.898 nm, as measured by DLS assay (Fig. 3B). To improve the chemical stability for *in vitro* and *in vivo* applications, 2’-F modified C and U nucleotides were applied to the RNA nanoparticles, resulting in significant resistance to enzymatic degradation. In addition, the melting temperature (T_m_) of FA-3WJ-PI-103 was determined to be approximately 62.5°C in a TGGE (Fig. 3C,D), which demonstrates that FA-3WJ-PI-103 can remain thermodynamically stable in physiological environments and at low concentrations.

Next, 3WJ-PI-103 nanoparticles were incubated in low pH conditions to confirm drug release from fully assembled 3WJ nanoparticles at low pH and to determine whether drug release from 3WJ nanoparticles at low pH achieved a drug concentration gradient enough to inhibit PI3K/mTOR pathway *in vitro*. First, we determined the drug concentration required to achieve PI3K/mTOR pathway inhibition after direct treatment with PI-103 diluted in DMSO. HCT116 and HT29 cells were treated with PI-103 diluted in DMSO at 1, 2.5, 5, 10 and 25 μM for 4, 12 and 24 h. PI-103 treatment resulted in rapid and long-lasting inhibition of PI3K in HCT116 cells; PI3K inhibition in HT29 cells was short-term and required a 5x higher dose of PI-103 to achieve compared to HCT116 cells (Supplemental Fig. 4A,B). Caco-2, JAR and SK-OV-3 cells were sensitive to PI-103 treatment and achieved complete PI3K inhibition at a dose of 5 μM (Supplemental Fig. 4C). PI-103 was released from 3WJ-PI-103 nanoparticles after incubation in low pH conditions and added to HCT116 cells cultured in complete media at 5 μM concentration. Short-term incubation of 3WJ-PI-103 nanoparticles at low pH confirmed quick drug release and drug activity, verifying that PI-103 function is preserved during the 3WJ nanoparticle assembly process. (Fig. 3E). Next, PI-103 was released from 3WJ-PI-103 nanoparticles after incubation at pH 3; HCT116 cells were treated at dosages of 0.5, 1, 1.5, 2, 2.5 and 5 μM to compare to the dosage range used with direct treatment of PI-103 diluted in DMSO. As shown in Fig. 3F, drug release from 3WJ-PI-103 is sufficient to achieve a similar level of PI3K inhibition as observed after direct treatment with PI-103 diluted in DMSO. Our experiments with *ex vivo* incubation of 3WJ-PI-103 in a low pH environment were an important step to demonstrate rapid drug release from 3WJ-PI-103 that leads to efficient suppression of molecular signaling in cancer cells, but *ex vivo* experiments cannot confirm internalization of 3WJ-PI-103 nanoparticles by FRα-FA-3WJ interaction and drug escape from lysosomes.

### FA-3WJ-PI-103 nanoparticle internalization and drug release in CRC cells and PDX lines

**Fig. 4.**
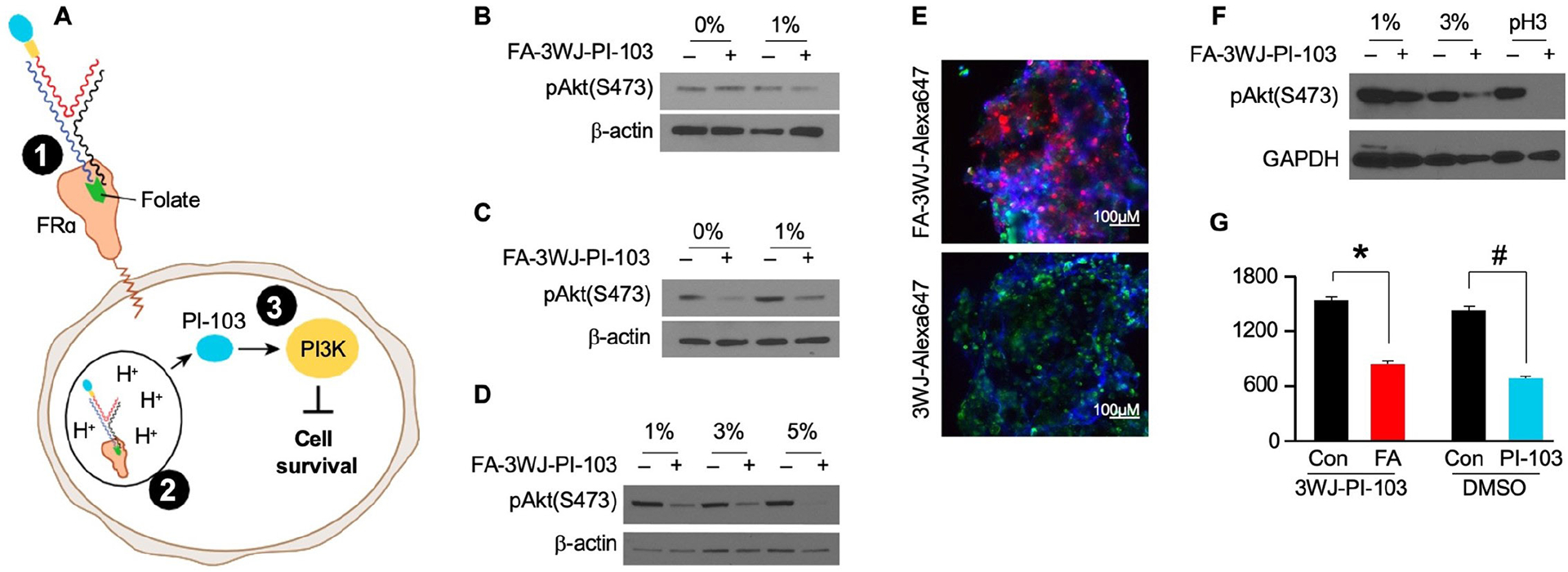
FRα mediated binding, internalization, drug release and PI3K inhibition from FA-3WJ-PI-103 nanoparticle. **(A)** Diagram of receptor mediated drug delivery steps required for PI3K inhibition. **(B)** HCT116 cells were treated with 5 μM FA-3WJ-PI-103 in 400 μl of 0% or 1% FA- complete media for 4h; cell culture media was replaced with 10% FA-complete media and evaluated for pAkt (Ser473) expression 4h later. **(C)** HCT116 cells were cultured in 10% FA- complete media for 7 days, plated into 24-well plate in FA- complete media and treated next day with 5 μM FA-3WJ-PI-103 in 400 μl of 0% or 1% FA- complete media for 4h; cell culture media was replaced with 10% FA-complete media and evaluated for pAkt (Ser473) expression 4h later. **(D)** HCT116 cells were cultured in 10% FA- complete media for 7 days, plated into 24-well plate and treated next day with 5 μM FA-3WJ-PI-103 in 400 μl of 1%, 3% and 5% FA- complete media for 2h; cell culture media was replaced with 10% FA-complete media and evaluated for pAkt (Ser473) expression 3h later. (**E**) PDX 2387 organoids were cultured in F- complete media for 7 days in ultra-low attachment 24-well plate. Spheroids were treated with 500 nM FA-3WJ-Alexa647 and 3WJ-Alexa647 nanoparticles in 500 μl of 1% F- complete media for 4h; binding and internalization of fluorescent nanoparticles was analyzed with confocal microscopy; blue for nuclear DNA: DAPI; magenta for 3WJ particles: Alexa647; green for F-actin filaments: Alexa Fluor^™^ 488 phalloidin; magnification 20x. **(F)** PDX2387 cells were cultured in FA- complete media for 7 days and treated with 10 μM FA-3WJ-PI-103 in 400 μl of 1% and 3% FA- complete media for 2h; cell culture media was replaced with 10% FA-complete media and evaluated for pAkt (Ser473) expression 3h later. FA-3WJ was used as a control; incubation of FA-3WJ-PI-103 *ex vivo* at pH3 was used as a positive control for rapid drug release from 3WJ nanoparticles at low pH. **(G)** PDX2387 cells were cultured in FA- complete media for 7 days and plated into 96-well plate at 5000 cells per well. Cells were treated next day with 10 μM FA-3WJ-PI-103, 3WJ-PI-103 or PI-103 diluted in DMSO in 100 μl of 5% FA- complete media; CCK8 assay was performed 48h after treatment; *p<0.05 compared to 3WJ-PI-103 control; #p<0.05 compared to DMSO control.

At this stage in the study, we have demonstrated FRα-FA-3WJ interaction (Fig. 4A, ①) and drug release in an environment simulating lysosomal pH (Fig. 4A, ②). Next, we performed a series of experiments to confirm PI3K inhibition after direct treatment with FA-3WJ-PI-103 nanoparticles (Fig. 4A, ③). Here, PI3K inhibition again served as a sensitive readout to demonstrate that FA-3WJ-PI-103 nanoparticles bind to FRα, internalize into endosomes and that PI-103 is released at low pH from 3WJ nanoparticles to inhibit the PI3K pathway. As a next step, we examined whether the presence of FA in media or FA depletion is necessary for FA-3WJ-PI-103-targeted receptor-mediated intracellular delivery by endocytosis.

HCT116 cells without FA depletion were treated with 5 μM FA-3WJ-PI-103 in 400 μl of 0% or 1% FA-free complete media for 4 h. Cell culture media was replaced with 10% FA-free complete media and evaluated for pAKT(Ser473) expression 4 h later. There was no inhibition of pAKT observed in cells treated in the FA-rich environment (Fig. 4B). Next, we repeated the treatment in cells depleted of FA by culturing in FA-free 10% FBS media; HCT116 cells were cultured in 10% FA-free media for 7 days and then treated with 5 μM FA-3WJ-PI-103 in 400 μl of either 0% or 1% FA-free complete media for 4 h. Cell culture media was replaced with 10% FA-free complete media and evaluated for pAKT(Ser473) expression 4 h later. FA depletion in media and cancer cells enhanced FA-3WJ-PI-103 internalization as shown by PI3K inhibition (Fig. 4C). Next, we examined the stability and function of FA-3WJ-PI-103 at higher concentrations of serum and demonstrated that an increase in serum concentration did not affect the stability of FA-3WJ-PI-103 nanoparticles and resulted in PI3K inhibition from direct treatment with FA-3WJ-PI-103 nanoparticles. HCT116 cells were incubated in 1%, 3% and 5% media for 2 h with inhibition of PI3K pathway activation noted in all treatment groups (Fig. 4D). We then examined FRα-mediated RNA nanoparticle uptake in a PDX cell line tumor spheroid model system. PDX2387 tumor spheroids were treated with FA-3WJ-Alexa647 nanoparticles in FA-free 1% cell culture media (Fig. 4E). Confocal imaging confirmed fluorescent nanoparticle accumulation in PDX2387 tumor spheroids at 4 h. PDX2387 treatment with FA-3WJ-PI-103 nanoparticles resulted in pAKT(Ser473) inhibition and suppression of cancer cell proliferation (Fig. 4F, G).

**Fig. 5.**
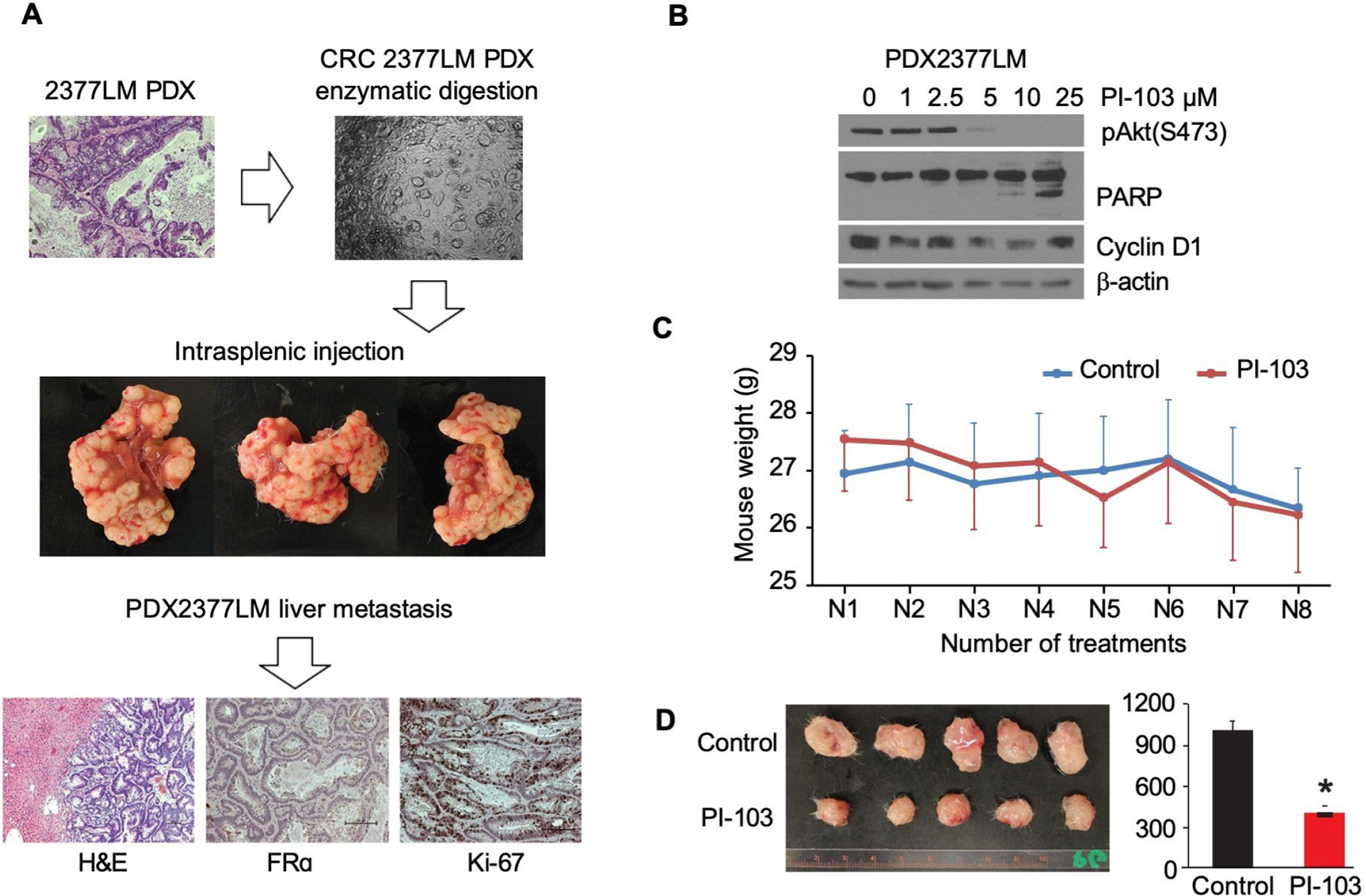
CRC 2377LM subcutaneous tumors treatment with PI3K/mTOR inhibitor PI-103. **(A)** PDX cell line was established from patient F3 CRC lung metastasis PDX tumor sample. PDX2377LM cells were injected intrasplenic into NOD-scid IL2Rgammanull mice; all mice developed liver metastasis after cancer cell injection. **(B)** 2377LM cell were treated with PI-103 at 1, 2.5, 5, 10, 25 μM for 24h. **(C)** Mouse weight was measured for both groups treated with PI-103 and vehicle (30 mg/kg; diluted in DMSO; ip; twice a day) for 7 days. **(D)** Measurement of subcutaneous tumors volume (mm^3^) with calipers at the end of the experiment.

In the last section of our study, we evaluated the therapeutic potential of PI3K/mTOR inhibitor PI-103 in CRC therapy. Resected CRC liver metastases were used to establish PDX tumors 2377LM and 2647LM; 2377LM cell line was isolated from PDX tumor and analyzed by IHC for FRα receptor expression and Ki-67 proliferation marker expression (Fig. 5A; Supplemental Fig. 5). PI3K inhibition, after PI-103 treatment *in vitro*, was confirmed in the 2377LM cell line. At high doses, PI-103 treatment induced cancer cell apoptosis as observed by cleaved PARP and cell cycle inhibition with cyclin D1 suppression (Fig. 5B). Mice were treated in vivo at 30 mg/kg dose with PI-103 diluted in DMSO administered intraperitoneally twice a day, q3d. PI-103 treatment of 2377LM subcutaneous tumors or 2647LM PDXs confirmed selective sensitivity of CRC to PI-103 therapy. Treatment with PI-103 suppressed growth of 2377LM tumors, but not 2647LM PDX tumors (Fig. 5C,D, Supplemental Fig. 5).

Our *in vitro* results demonstrate that RNA molecules can be programmed to function as pH-responsive small-molecule cargo carriers. Moreover, we demonstrate the benefit of a specific signaling pathway inhibitor conjugated to 3WJ nanoparticles, compared to standard chemotherapy agents. The PI3K/mTOR inhibitor served a dual role—as a therapeutic agent and as a tool for rapid confirmation of successful drug delivery into cancer cells. By monitoring the activity of AKT protein kinase as a readout for FRα-mediated drug delivery by 3WJ nanoparticles, we confirmed that PI-103 remains fully functional and stable over the course of receptor-targeted small molecule drug delivery to cancer cells. Therefore, we successfully demonstrated use of PI-103 as a scientific tool in the exploration of drug delivery by RNA nanoparticles.

In summary, we developed a pH-responsive drug delivery vehicle for receptor-selective drug delivery to cancer cells with high FRα expression and identified guidelines for selection of candidate PI3K/mTOR inhibitors for treatment of cancers sensitive to aberrant PI3K signaling inhibition.

## DISCUSSION

The success of colorectal cancer therapy depends on several key factors, including sensitivity of cancer cells to a small molecule drug, concentration of a small molecule drug in cancer tissues, and the patient’s ability to endure on-target side effects of normal tissues^38, 39^. The main drawbacks of conventional administration of small molecular inhibitors or chemotherapeutic agents are high dose requirements, low therapeutic indices and permanent damage to healthy tissues^39, 40^. Therefore, receptor-targeted delivery of small molecule drugs is a promising approach to increase the concentration of a chemotherapeutic agent or small molecule inhibitors in cancer tissues and to minimize normal organ damage.

RNA nanoparticles are ideal for receptor-mediated intracellular drug delivery due to absence of immune response, specific cell surface receptor targeting and a predictable size of a construct ranging from 10 to 50 nm^41–43^. Fusion of a targeting ligand (e.g., FA) with one of the 3WJ strands produces a thermodynamically stable 3WJ-aptamer chimera structure with high binding affinity to cell surface receptors^44^. FA-3WJ nanoparticle binding to FRα receptor results in internalization through the endocytic pathway into intracellular lysosomes with pH values as low as 4.0–4.5^45–48^. Therefore, efficient delivery of therapeutic drugs depends on both nanoparticle recognition of specific cellular receptor and the ability of drug delivery systems to escape the endosome-lysosome pathway initiated upon cellular uptake. This process begins with internalization into an endocytic vesicle, followed by fusion with the early endosome, progression into the late endosome, and final maturation into the lysosome. Throughout the transition from endosome to lysosome, the pH gradually decreases from a physiological 7.4 to around 5.0 within the lysosome^49–51^. Failure to rapidly release drugs from RNA particles in mature lysosomes can lead to their entrapment and potential degradation, rendering the delivery unsuccessful. Once in lysosomes, the success of drug delivery by 3WJ nanoparticles is dependent on pH-sensitive drug linker decomposition and sustained drug release of therapeutic agents at the low pH environment of lysosomes. Acid-labile drug linker is stable at pH 7.2–7.4, but quickly undergoes cleavage at low pH^49–51^. We introduced an acid labile linker between the 3WJ nanoparticle and PI-103 to facilitate a controlled release of drug in the low pH environment of the endosome. Functionalization of FA on RNA nanoparticles and the addition of an acid labile linker significantly reduced chances of drug release in normal tissues with low FRα expression.

A critical step in our study was the selection of a small molecule drug for conjugation. The primary objective of this study was to confirm receptor-mediated intracellular drug delivery and drug release from endosomes. Successful intracellular small molecule delivery and release from lysosomes leads to rapid inhibition of cell signaling compared to a delayed effect associated with traditional chemotherapeutic agents. Monitoring effects from intracellular delivery of traditional chemotherapeutic agents, such as 5-FU, SN-38 or gemcitabine, relies on measuring the indirect effect of a chemotherapy agent on cancer cell proliferation at long timepoints^52^. For that reason, we decided to use a small molecule inhibitor with rapid action on intracellular signaling. AKT phosphorylation was used as a reliable and specific readout of PI3K inhibition after FRα-mediated drug delivery by 3WJ nanoparticles. We successfully delivered the PI3K/mTOR inhibitor into cancer cells. However, our experiments *in vitro* and *in vivo* also identified that, as a therapeutic agent, PI-103 is not suitable for conjugation to RNA nanoparticles due to the resistance of CRC cells to PI-103 therapy and a requirement for a high dose of PI-103 to suppress CRC proliferation *in vivo*.

The results from our study led to formulation of PI3K small molecule inhibitors selection guidelines for conjugation to RNA nanoparticles: 1) conjugation drug candidates must demonstrate high activity in the nM range; 2) broad sensitivity of cancer cells to a select agent; 3) long duration of pathway inhibition; and 4) drug stability in extracellular space *in vivo*. This selection criteria are also useful when applied to chemotherapy agents. For example, SN-38 demonstrates high activity in nM range, broad sensitivity of cells to the agent and stability *in vivo*. We demonstrated SN-38 chemotherapy drug delivery with RNA nanoparticles against CRC lung metastases and showed that RNA nanoparticles harboring 2’-F modified pyrimidine and 24 copies of SN-38 efficiently suppressed CRC metastasis progression *in vivo*^58, 59^.

The PI3K/mTOR activity is important for survival during stress^53, 54^. Our earlier studies showed that PI3K/mTOR inhibition during the cellular stress of metastasis or during radiation therapy enhanced the therapeutic potential of PI3K/mTOR inhibition and leads to cancer cell apoptosis^55, 56^. Our results *in vivo* demonstrate that use of PI3K/mTOR inhibitors alone are effective at suppression of cancer cells proliferation, but ineffective as a method for elimination of cancer cells. For example, we showed that the delivery of the panPI3K inhibitor, PX866, by polymeric micelles suppressed the proliferation of CRC lung metastases; however, only combination therapy with the chemotherapeutic agent, SN-38, completely eliminated CRC lung metastases^57^. Therefore, full potential of PI3K/mTOR inhibitors can be achieved in combination with chemotherapy agents or radiation therapy. We envision that conjugation of two different therapeutic agents to branches of the same 3WJ nanoparticle for a combinational effect of PI3K/mTOR inhibitor and a chemotherapy agent would greatly enhance therapeutic effect and serve as a viable strategy to eliminate cancer cells.

## CONCLUSION

In summary, we demonstrate receptor-mediated uptake of FA-pRNA-PI-103 nanoparticles and inhibition of the PI3K/AKT pathway in CRC cells and tumor spheroids. These results confirm conjugation of a drug to a fully-assembled 3WJ RNA nanoparticle with preservation of pH-selective drug release and receptor-mediated drug delivery into CRC cells. The unique targeting abilities of pRNA in combination with small-molecule drugs offers a novel strategy for receptor-selective drug delivery to cancer cells and has the potential to enhance the efficacy of anti-cancer therapies while mitigating dose-limiting toxicity in patients with CRC.

## Supplementary Material

1

## ABBREVIATIONS:

ANOVA: analysis of variance
CRC: colorectal cancer
CuAAC: copper(I)-catalyzed azide-alkyne cycloaddition
DMSO: dimethyl sulfoxide
DLS: dynamic light scattering
FRα: folate receptor alpha
FA: folic acid
IHC: immunohistochemistry
pRNA: packaging RNA
PDX: patient-derived xenograft
PI3K: phosphoinositide 3-kinase
RFC: reduced folate carrier
ssRNA: single-stranded RNA
TGGE: temperature gradient gel electrophoresis
3WJ: three-way junction
