# Supplementary material for: pH-responsive bond as a linker for the release of chemical drugs from RNA–drug complexes in endosome or lysosome
Source: RNA Nanomed. Author manuscript; Available in PMC 2025 Mar 20. (PMC11925213; doi:10.59566/isrnn.2024.0101091)
Supplement: 1 [file NIHMS2063703-supplement-1.pdf]

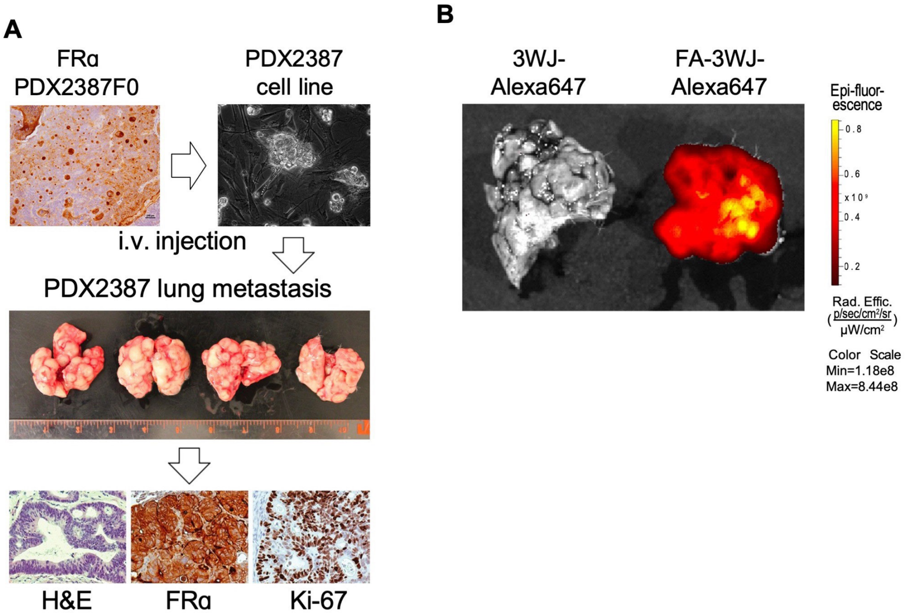

Supplemental Fig. 1. FA-3WJ-Alexa647 nanoparticle binding to colorectal cancer PDX-derived cell line FRα. (A) PDX cell line was established from patient F3 CRC lung metastasis PDX tumor sample. PDX2387 cells were injected iv into NOD-scid IL2Rgammanull mice; all mice developed lung metastasis 5 month after cancer cell injection. IHC analysis confirmed high FRα expression in F0 PDX tumor sample and preservation of FRα expression in metastatic tumors. **(B)** NOD SCID mice were injected intravenously with PDX2387 cell line (100 µl, 1x10^6) and placed on a folate free diet. Three weeks after cancer cells injection mice were treated with 3WJ-Alexa647 and FA-3WJ-Alexa647 pRNA every 30 min (3 treatments; 1.2 µg/g; 500 µl; pRNA diluted in PBS). Thirty minutes after last pRNA injection lungs were collected and imaged on IVIS Spectrum.

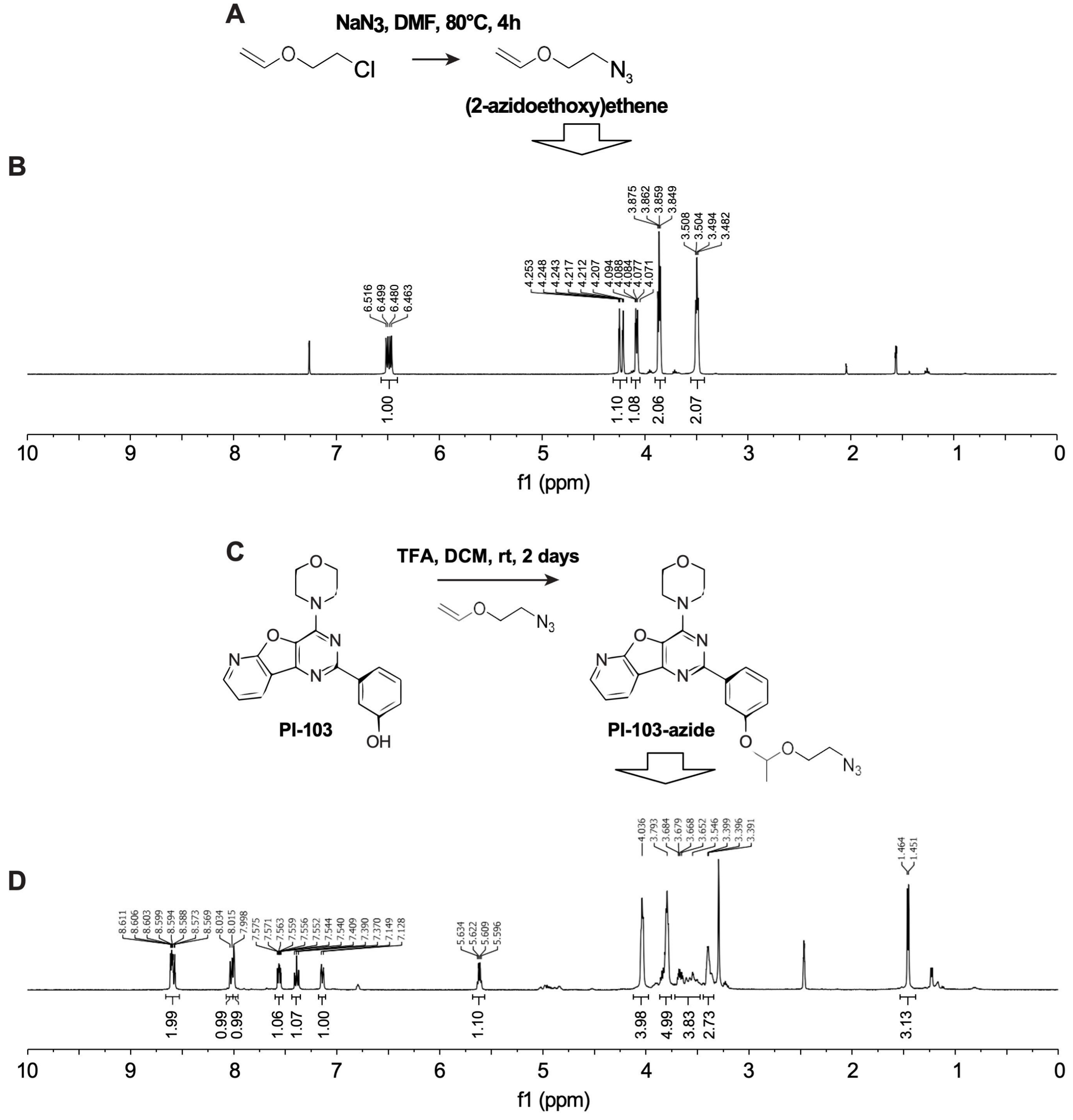

Supplemental Fig. 2. Synthesis of the precursor. (A). Diagram of (2-Azidoethoxy)ethene synthesis. **(B).** 1H-NMR spectrum (CDCl3, 400 MHz) of (2-azidoethyoxy)ethene. **(C).** Diagram of 2-(3-(1-(2-Azidoethoxy)ethoxy)phenyl)-4-morpholinopyrido[3',2':4,5]furo[3,2-d]pyrimidine (PI103-azide) synthesis. **(D)**. 1H-NMR spectrum (DMSO-d6, 400 MHz) of PI-103-azide.

A

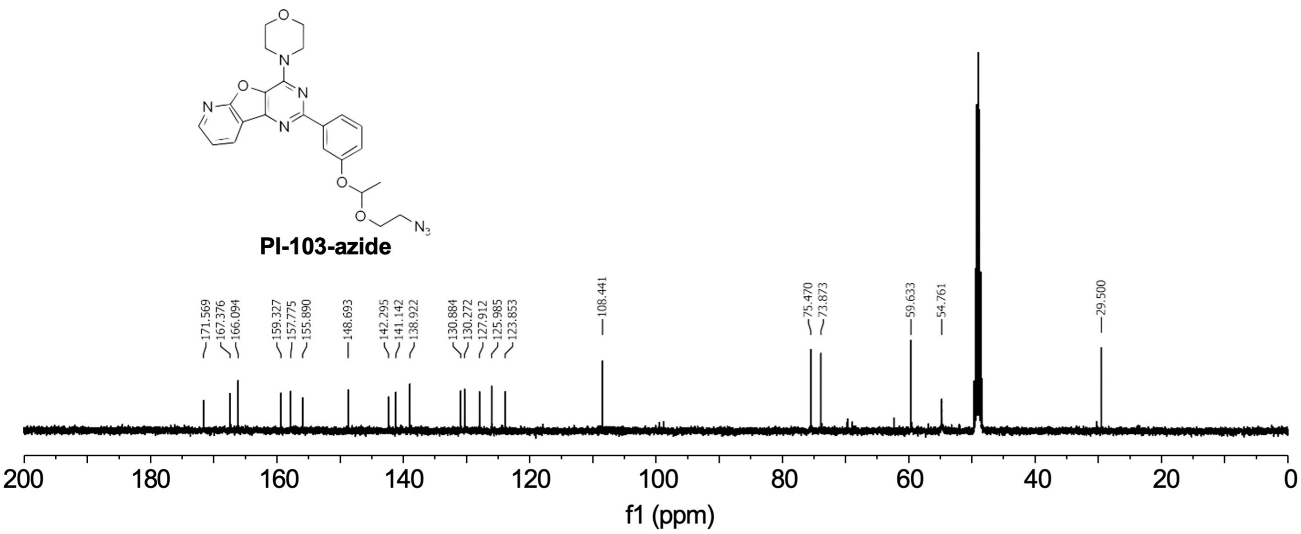

B

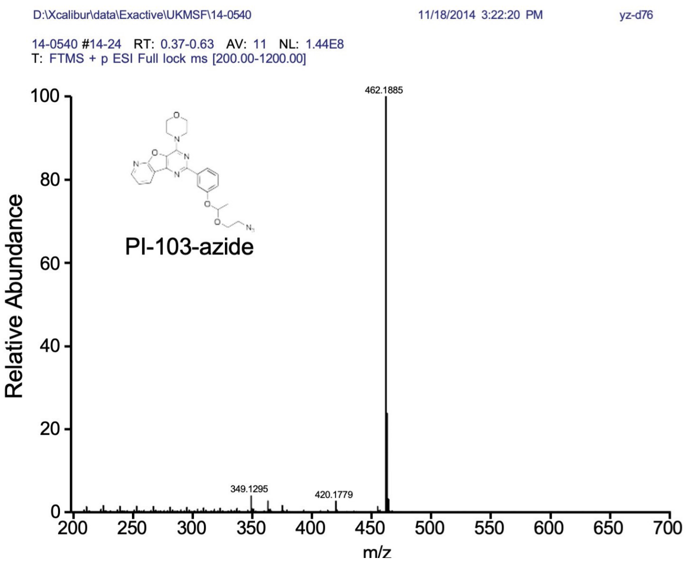

C

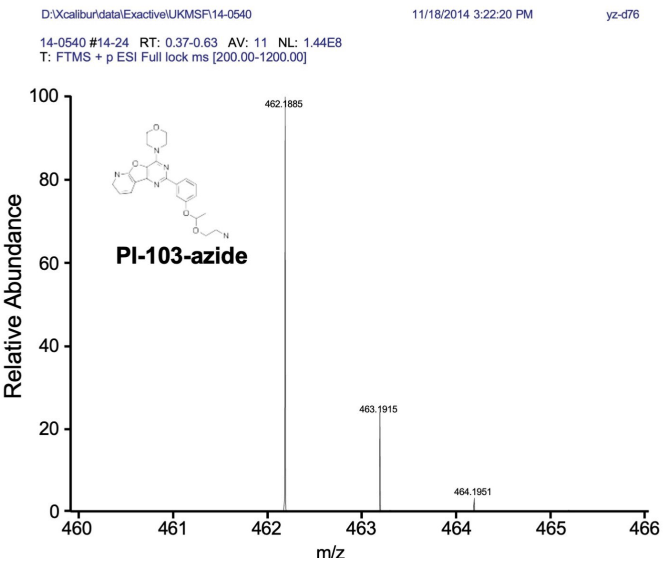

Supplemental Fig. 3. Nuclear magnetic resonance spectroscopy of PI-103-azide. (A).^{1}H-NMR spectrum (CD_3OD, 100 MHz) of PI-103-azide. **(B).** HR-MS spectrum of PI-103-azide.

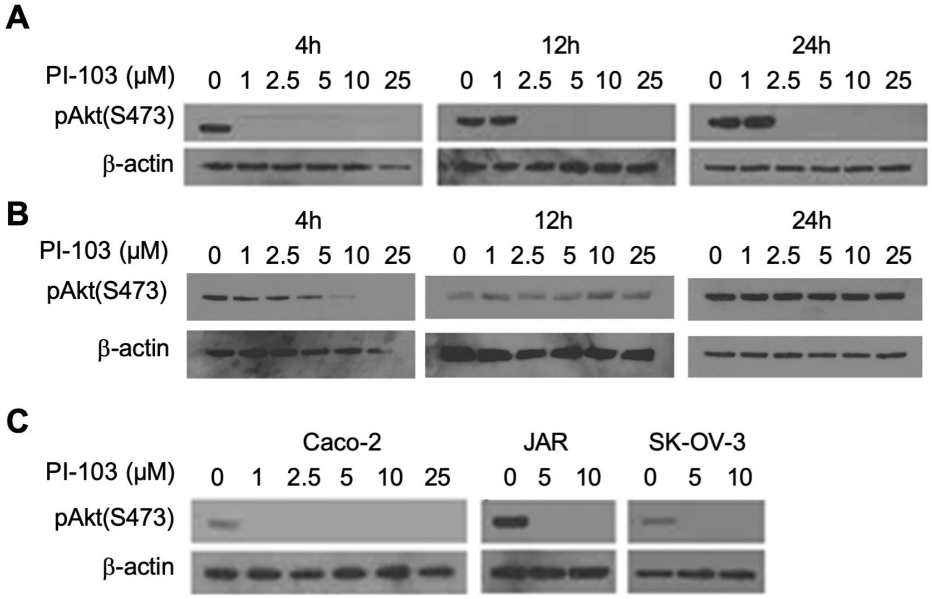

Supplemental Fig. 4. Cancer cell lines sensitivity to PI-103 treatment. (**A**). HCT116 cells were treated with PI-103 dilute in DMSO at 1, 2.5, 5, 10, 25 μM for 4, 12 and 24h. **(B)**. HT29 cells were treated with PI-103 diluted in DMSO at 1, 2.5, 5, 10, 25 μM for 4, 12 and 24h. **(C).** Caco-2, JAR and SK-OV-3 cells were treated with PI-103 diluted in DMSO for 24h.

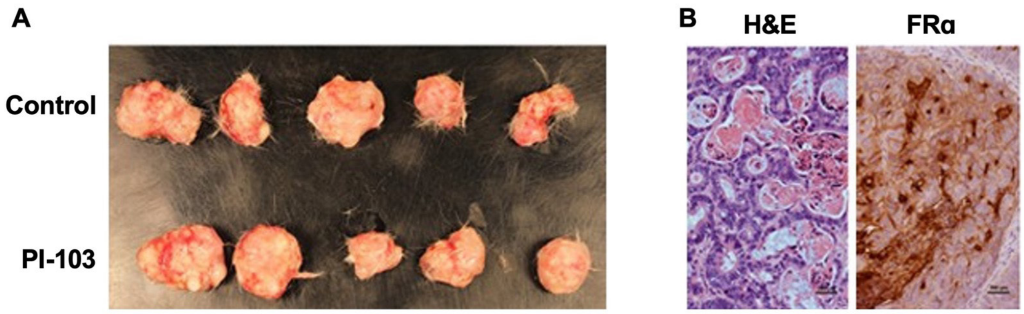

Supplemental Fig. 5. **CRC 2647LM subcutaneous PDX tumors treatment with PI3K/mTOR inhibitor PI-103. (A).** NOD SCID mice were transplanted with gen2 PDX 2647LM. Four weeks after tumor implantation mice were treated with PI-103 and vehicle (30 mg/kg; diluted in DMSO; ip; twice a day, q3d) days for 8 wks**. (B).** H&E and FRα IHC analysis of CRC2647LM PDX tumor.
